# Degradation of antifungal anthraquinone compounds is a probable physiological role of DyP secreted by *Bjerkandera adusta*

**DOI:** 10.1186/s13568-019-0779-4

**Published:** 2019-04-23

**Authors:** Kanako Sugawara, Etsuno Igeta, Yoshimi Amano, Mayuko Hyuga, Yasushi Sugano

**Affiliations:** 1 Department of Chemical and Biological Sciences, Faculty of Science, Japan Women’s University, 2-8-1 Mejirodai, Bunkyo-ku, Tokyo, 112-8681 Japan; 2 Division of Material and Biological Sciences, Graduate School of Science, Japan Women’s University, 2-8-1 Mejirodai, Bunkyo-ku, Tokyo, 112-8681 Japan

**Keywords:** DyP, Dye-decolorizing peroxidase, Basidiomycetes, *Bjerkandera adusta*, Antifungal anthraquinone compounds, Physiological role

## Abstract

Alizarin is an anti-fungal compound produced by the plant, *Rubia tinctorum*. The parasitic fungus *Bjerkandera adusta* Dec 1 was cultured in potato dextrose (PD) medium with or without alizarin. Alizarin was a good substrate for the dye-decolorizing peroxidase (DyP) from *B. adusta* Dec 1 and hampered *B.* *adusta* growth at the early stage of plate culture. During liquid shaking culture, DyP activity in cultures supplemented with 100 μM alizarin was greater than that in controls cultured without alizarin. In particular, DyP activity per dry cell mass increased approximately 3.5-, 3.1-, and 2.9-fold at 24, 30, and 36 h after inoculation, respectively, compared with control cultures. These data suggest that alizarin stimulates the expression of DyP. Interestingly, alizarin rapidly decomposed at an early stage in culture (24–42 h) in PD medium supplemented with 100 μM alizarin. Thus, alizarin appears to induce DyP expression in *B. adusta* Dec 1, and this DyP, in turn, rapidly degrades alizarin. Collectively, our findings suggest that the physiological role of DyP is to degrade antifungal compounds produced by plants.

## Introduction

*Bjerkandera adusta*, a basidiomycete belonging to the family Polyporaceae, is a white rot fungus that parasitizes certain trees, resulting in lignin degradation. Notably, *B. adusta* Dec 1 (FERM P-15348) decolorizes kraft pulp lignin (Shintani et al. 2002). Manganese peroxidase (MnP) and dye decolorizing peroxidase (DyP) have been detected during culture of *B. adusta* Dec 1, but lignin peroxidase (LiP) and laccase have not. Another noteworthy characteristic is that Dec 1 degrades xenobiotics such as synthetic anthraquinone dyes and secretes a versatile peroxidase (VP) in addition to DyP during culture (Kim et al. 1995; Sugano et al. 2000, 2006, 2009; Gomi et al. 2011). However, VP activities toward several anthraquinone compounds are only ~ 2–20% of those of DyP, clearly indicating that DyP of *B. adusta* is the main degrader of anthraquinone compounds (Sugano et al. 2006). On the other hand, because synthetic dyes are never true substrates, the physiological role of DyP is unknown and thus remains an essential question.

DyP of *B. adusta* is a member of a large family of DyP-type peroxidases that is subdivided in three classes, P, I and V, according to their tertiary structural homology (Yoshida and Sugano 2015). A unique feature of this family is that their characteristics, including cellular localization and primary structures, vary widely and differ considerably among the three classes (Sugano et al. 2007; Sugano 2009; Yoshida and Sugano 2015). Members of class P, which have the smallest molecular size among the three classes, are characterized by general peroxidase activity, but low anthraquinone degradation activity (Ahmad et al. 2011; Roberts et al. 2011; Singh et al. 2012; Rahmanpour and Bugg 2013; Yoshida and Sugano 2015). Members of class I are intermediate in size, and some show moderate decolorizing activity toward anthraquinone dyes (Ahmad et al. 2011; Roberts et al. 2011; Santos et al. 2014). Of the three classes, members of class V, which are distributed among both prokaryotes and eukaryotes, have the largest molecular size (Yoshida and Sugano 2015; Sugawara et al. 2017). Notably, most members of class V are produced and secreted by basidiomycetes (Johjima et al. 2003; Scheibner et al. 2008; Liers et al. 2010, 2013; Kellner et al. 2014), predominantly white rot fungi such as *B. adusta*, and show high decolorizing activity toward anthraquinone dyes (Sugano et al. 2000; Liers et al. 2010, 2013; Salvachúa et al. 2013). From another standpoint, the class V DyP-type peroxidases, AjP I, AjP II, EglDyP and MepDyP, from basidiomycetes are secreted outside the cell and degrade non-phenolic lignin model compounds through their peroxidase activity (Liers et al. 2010, 2013). This suggests that these proteins function in the oxidative degradation of lignin. Consistent with this, widespread transcript-level expression of DyP-type peroxidases has been confirmed in almost all samples of fungi from forest floor habitats (Kellner et al. 2014). However, the lignin-degrading activity of DyP-type peroxidases from basidiomycetes is at most 4% of that of LiP from *Phanerochaete chrysosporium* (Liers et al. 2013; Linde et al. 2015). DypB from *Rhodococcus jostii* has been reported to degrade lignin in the presence of Mn(II), but its activity is low compared with that of fungal lignin-degrading enzymes (Ahmad et al. 2011; Brown et al. 2012; Linde et al. 2015).

In contrast, LiP, MnP, and VP are well known as major contributors to lignin degradation in white rot fungi (Wariishi et al. 1989; Camarero et al. 1999; Johjima et al. 1999; Pollegioni et al. 2015). Actually, some basidiomycetes that express LiP and MnP, but lack DyP-type peroxidases, such as *P. chrysosporium*, exhibit high lignin-degradation activity (Korripally et al. 2015). These observations suggest the possibility that the true physiological role of DyP from basidiomycetes is something other than lignin degradation, prompting us to focus on anthraquinone compounds as potential substrates. As mentioned above, DyP readily degrades anthraquinone compounds (Sugano et al. 2000; Ogola et al. 2009; Liers et al. 2010, 2013; Salvachúa et al. 2013). Interestingly, plants, including trees, express a number of anthraquinone compounds that serve antifungal functions. One such representative antifungal compound is alizarin, produced by the evergreen perennial, *Rubia tinctorum* (Manojlovic et al. 2005; Jara et al. 2017). Generally, white rot fungi belonging to the class basidiomycetes are best known for their selective parasitism of old or dead trees. They rarely grow on young or healthy trees because these trees generate phytoalexin, which serves to protect against infection (Wijnsma et al. 1985). In contrast, *B. adusta* is often observed to parasitize some living trees in a forest (Berry and Lombard 1978). This raises the question of how *B. adusta* evades the defense of plants.

In this study, we focused on the ability of DyP to degrade the anti-fungal anthraquinone compound, alizarin (Manojlovic et al. 2005), and further considered the possibility that DyP degrades natural anthraquinone compounds, such as some phytoalexins (Wijnsma et al. 1985). If this were true, it would help explain how some basidiomycetes parasitize living trees, despite the fact that these trees produce antifungal compounds, such as anthraquinones (Amaral et al. 2013). Here, we found that alizarin stimulated the secretion of DyP by the white rot fungus *B. adusta*. This is the first report that DyP truly degrades an antifungal anthraquinone compound in plants, a finding that could set the stage for resolving questions regarding interactions between fungi and plants.

## Materials and methods

### Microorganisms, media, DyP, and chemical reagents

The spores of *B. adusta* Dec 1, previously isolated by us, were kept in 25% glycerol at − 80 °C (Kim et al. 1995). Potato dextrose (PD) medium was prepared as described previously (Sugano et al. 2006). Purified recombinant DyP in *Aspergillus oryzae* was prepared using a previously reported method (Sugano et al. 2000). Alizarin and Remazol brilliant blue R (RB19) were purchased from Wako Chemical Co. (Tokyo, Japan). All other reagents were of analytical grade unless otherwise specified.

### Culture

A suspension of *B. adusta* Dec 1 spores was inoculated onto PD agar plates (10 μl/plate) and incubated for 12 days at 29 °C. Mycelia from six plates were collected and suspended in 10 ml of sterilized, distilled water. The suspension was agitated vigorously for 7 min and filtered through gauze, after which 8-ml aliquots of filtrate, which included 1.0 × 10^7^–10^8^ spores/ml, were inoculated into 250 ml of PD containing 100 μM alizarin in shaking flasks. Alizarin was excluded from medium in control cultures. Cultures were grown at 29 °C with shaking at 140 rpm.

### Growth rate of *B. adusta* Dec 1 under plate culture conditions

PD agar plates (8.5 cm *ϕ*) containing five different alizarin concentrations (0, 1, 3.2, 10 and 32 μM) were prepared. A suspension of *B. adusta* Dec 1 spores was inoculated onto the center of each plate (10 μl/plate) and incubated at 29 °C. The diameters of mycelia on plates were measured periodically.

### Sampling and preparation of dry cell mass

Thirty milliliters of culture broth were periodically harvested from shaking flask cultures. The sampled broth was centrifuged at 1500×*g* (4 °C, 1 h) in conical tubes, and the supernatant and precipitate were separated. The collected supernatant was used to assay alizarin concentration and enzymatic activity.

A membrane filter method was used to measure dry cell mass. Briefly, after harvesting and centrifuging 30 ml of culture broth and separating the supernatant and precipitate as described above, the precipitates were re-suspended in 30 ml of 1 mM CaCl_2_ and then treated with 10 μl of α-amylase (Novozymes) at 70 °C for 1 h. Thereafter, the suspension was collected on a cellulose acetate membrane filter (pore size, 0.8 μm), which was subsequently dried at 80 °C for 24 h. The dry cell mass was then calculated from the total weight minus the weight of the membrane filter.

### HPLC analysis of alizarin

Saccharides in culture broth were removed by adding 1 ml of ethanol to 500 μl of the culture supernatant. The mixture was centrifuged at 4 °C (11,000×*g*, 10 min), after which the supernatant was filtered through a cellulose acetate membrane (pore size, 0.45 μm). The amount of alizarin in the resulting filtrate was measured by high-performance liquid chromatography (HPLC) using a Capcell Pak C8 column (4.6 mm *ϕ* × 250 mm) with a flow rate of 1.0 ml/min. Alizarin was eluted with a 45:55 (v/v) solution of 95% acetonitrile:20 mM ammonium formate (pH 3.0), and was detected at a wavelength of 254 nm.

### Assay of DyP activity

Thirty milliliters of supernatant from the culture broth was concentrated to 50 μl by ultrafiltration (exclusion size, 30 kDa), to which was added 450 μl of 25 mM citrate buffer (pH 5.5). The resulting solution was then re-concentrated to 50 μl. This operation was repeated twice, and the final volume was adjusted to 300 μl with the same buffer. This solution was prepared for assay of DyP activity at 30 °C by adding 15 μl of 20 mM RB19 (DyP substrate), 750 μl of 100 mM citrate buffer (pH 3.2), and 1920 μl of distilled water in a 3-ml cuvette. The reaction was initiated by adding 15 μl of 40 mM H_2_O_2_, and decolorization of RB19 was monitored spectrophotometrically at 593 nm. One unit of DyP activity was defined as the amount of enzyme required to decolorize 1 μmol of RB19 (ε_593_ = 9100 M^−1^cm^−1^) in the reaction mixture in 1 min at 30 °C.

### Degradation of alizarin by recombinant DyP

Alizarin is poorly soluble in water and thus was prepared as a 20 mM solution in dimethyl sulfoxide (DMSO). Reaction mixtures were prepared in a 3-ml cuvette by combining purified, recombinant DyP [(Sugano et al. 2000); final concentration, 1.55 nM], 18 μl of 20 mM alizarin (final concentration, 125 μM), 750 μl of 100 mM citrate buffer (pH 3.2) and 582 μl of DMSO (final concentration, 20%), and adjusting the total volume to 2985 μl with distilled water. The reaction was initiated by adding 15 μl of 40 mM H_2_O_2_, and decolorization was monitored spectrophotometrically at 427 nm. The alizarin-degrading activity of DyP was compared with that of RB19 under the same reaction conditions (i.e., containing 20% DMSO).

The optimum pH for recombinant DyP activity toward alizarin was determined using a citrate buffer (25 mM) covering a pH range of 3.0 to 5.5. Reaction conditions, except for buffer pH, were the same as above.

### Effects of pH on alizarin degradation in liquid cultures containing DyP

Twenty milliliters of culture broth and cells were periodically harvested from shaking flask cultures. The pH of culture broth was immediately measured and adjusted to 3.2. Thereafter, cultures were incubated at 30 °C for 30 min and centrifuged to remove cells. The alizarin concentration in the resulting supernatants was analyzed as described above. Controls were not pH-adjusted.

## Results

### Degradation activity of DyP toward alizarin

**Fig. 1:**
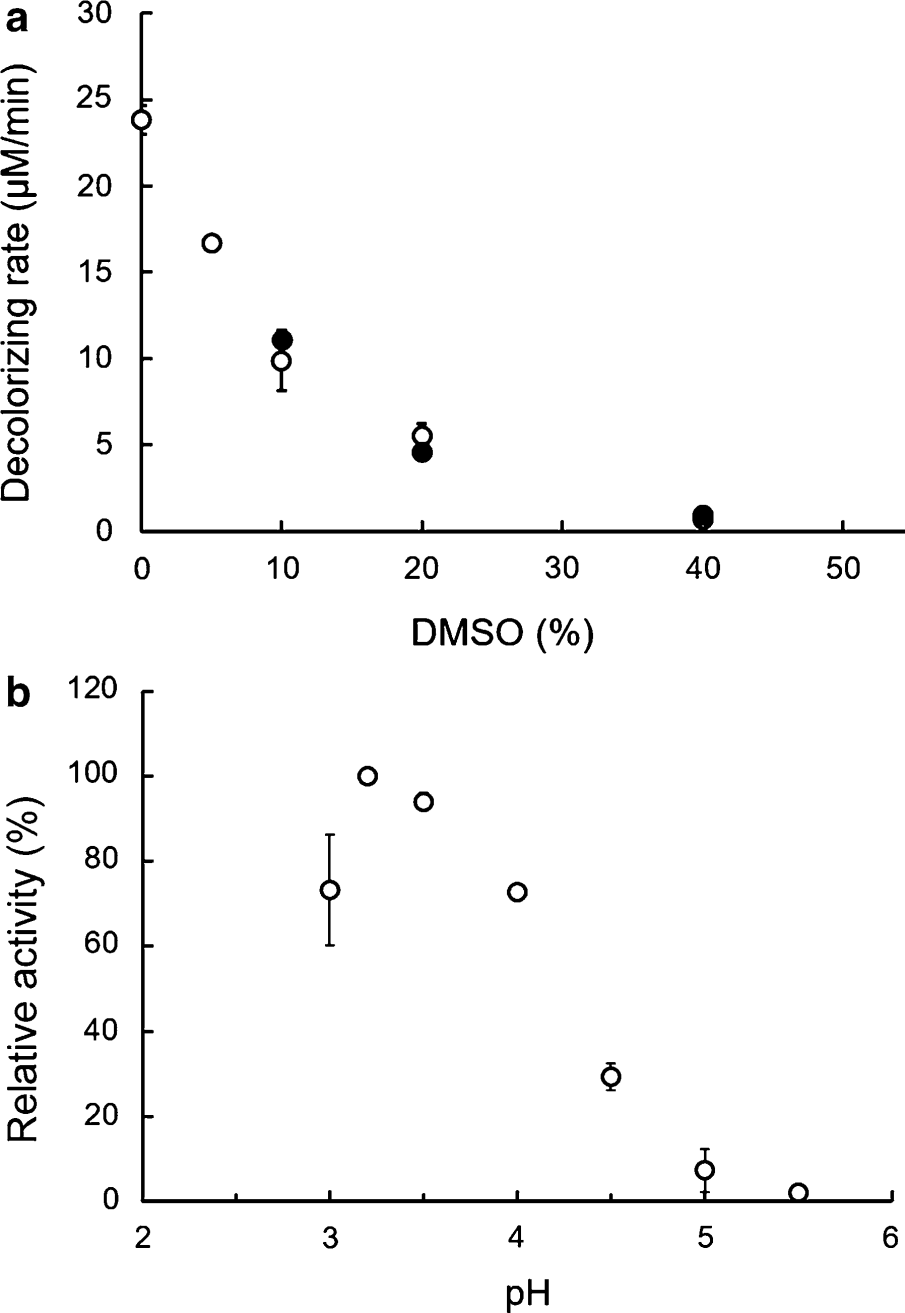
Effects of solvent and pH on DyP activity. **a** Relationship between DyP activity and DMSO concentration. Open and closed symbols denote the degradation activity of recombinant DyP towards RB19 and alizarin, respectively. **b** pH profile of the activity of recombinant DyP toward alizarin

At DMSO concentrations lower than 10%, alizarin did not completely dissolve in the reaction mixture. As shown in Fig. 1a, increasing the concentration of DMSO in the reaction mixture caused a gradual decrease in recombinant DyP activity. Notably, however, the degrading activity of DyP towards the general substrate RB19 and alizarin followed a similar pattern. For instance, in reaction solutions containing 20% DMSO, the degradation activity of DyP for RB19 and alizarin was 5.5 and 4.6 μM/min, respectively, indicating that both alizarin and RB19 are good substrates for DyP. An examination of the kinetic parameters of recombinant DyP toward alizarin were not able to yield *k*_cat_ values because of DMSO inhibition. As shown in Fig. 1a, DMSO in the reaction mixture reduced activity by up to 23% compared to the reaction without DMSO.

We also examined the optimum pH for degradation of alizarin by recombinant DyP. Recombinant DyP exhibited activity towards alizarin within the pH range of 3.0 to 5.0, and degraded most alizarin at pH 3.2 (Fig. 1b). These results are consistent with pH profiles for RB19 degradation activity, for which the reported optimum pH is 3.2 (Sugano et al. 2000).

### *B. adusta* Dec 1 growth in plate cultures

**Fig. 2:**
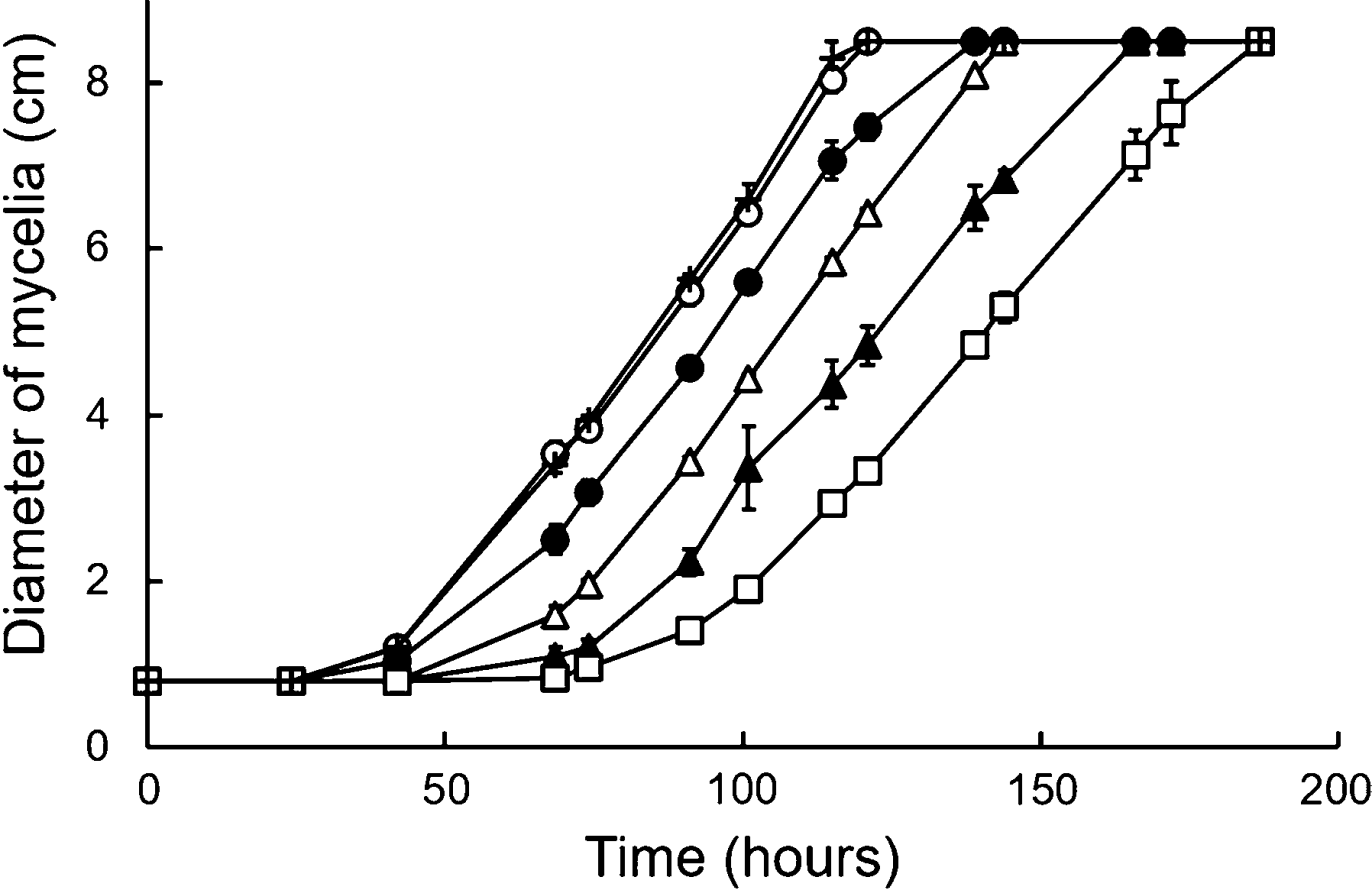
Time courses of *B. adusta* Dec 1 mycelia growth on PD plates containing different concentrations of alizarin. Crosses, 0 μM alizarin without DMSO; open circles, 0 μM alizarin with 0.64% DMSO; closed circles, 1 μM alizarin; open triangles, 3.2 μM alizarin; closed triangles, 10 μM alizarin; open squares, 32 μM alizarin

In initial experiments, we tested the effect of alizarin on *B. adusta* Dec 1 growth in plate cultures. As shown in Fig. 2, DMSO had no effect on *B.* *adusta* growth. After an initial lag phase (~ 25 h), *B. adusta* growth, measured as an increase in mycelia diameter, increased over time at all concentrations of alizarin tested. At a relatively early point after inoculation (69 h), the diameters of mycelia varied depending on the concentration of alizarin, with increasing concentrations of alizarin resulting in smaller diameters (Fig. 2). However, after 101 h, the growth rate (slope) was largely unaffected by alizarin concentration, suggesting that alizarin hampers growth at an early stage of plate culture, but not after middle stages of culture. Also, alizarin did not affect hyphal morphology in *B. adusta* Dec 1. Furthermore, the mycelia of *B. adusta* showed no change in color following culture with alizarin (orange-colored solution in DMSO), indicating that alizarin was not absorbed by mycelia.

### Time-dependent decrease in alizarin concentration in liquid shaking culture

**Fig. 3:**
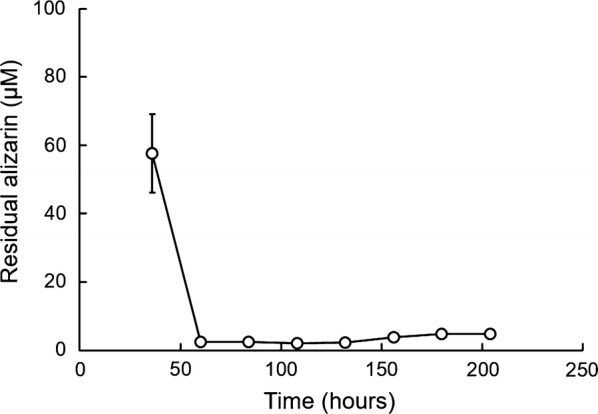
Time course of alizarin concentration in broth of liquid shaking cultures inoculated with 100 μM alizarin. Data are presented as mean ± SD (n = 3; *P < 0.05, unpaired two-tailed t test)

The time course of changes in alizarin concentration following inoculation with 100 μM is shown in Fig. 3. Interestingly, 36 h after inoculation, the alizarin concentration had decreased to 58 μM and stabilized at a very low level (< 5 μM) by 60 h. This indicates that alizarin was nearly completely degraded at an early stage in culture; if true, DyP must act efficiently at an early stage. In subsequent experiments, we sought to confirm this early-stage action, investigating effects within 48 h after inoculation with 100 μM alizarin.

### *B. adusta* growth in liquid culture

**Fig. 4:**
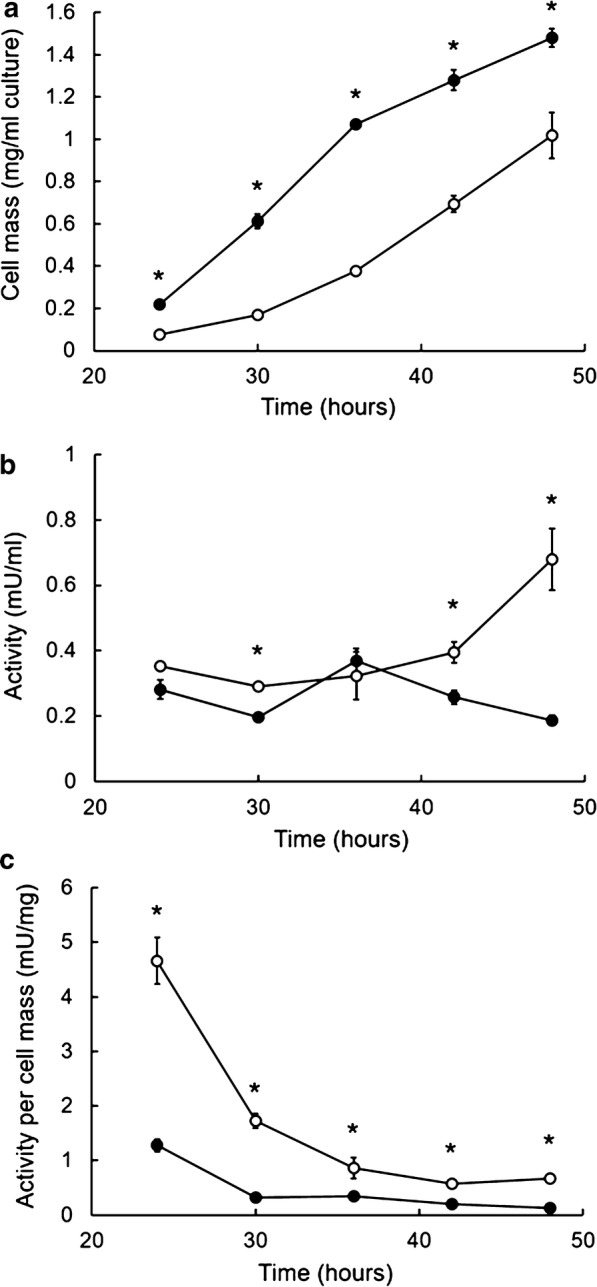
Time courses of dry cell mass accumulation and DyP activity at the early stage of culture with 100 μM alizarin. Open and closed symbols denote with and without alizarin, respectively. **a** Dry cell mass; **b** DyP activity per milliliter culture broth; **c** DyP activity per milligram dry cell mass. Data are presented as mean ± SD (n = 3; *P < 0.05, unpaired two-tailed t test)

To further assess the effect of alizarin on *B. adusta* growth, we measured cell mass accumulation over time in liquid cultures. A time course of cell mass accumulation following treatment with 100 μM alizarin is shown in Fig. 4a. Alizarin (100 μM) caused a decrease in dry cell mass compared with control cultures without alizarin at all sampling times.

### DyP activity in liquid culture

We next assessed DyP activity over time in liquid cultures of *B. adusta*, with or without alizarin. Time courses of DyP activity, expressed as milliunits per milliliter (mU/ml) of culture broth and milliunits per milligram (mU/mg) dry cell mass, are shown in Fig. 4b, c, respectively. Notably, DyP activity per cell mass in the presence of alizarin was approximately 3.5-, 3.1-, and 2.9-fold higher at 24, 30, and 36 h, respectively, than that observed in control cultures without alizarin (Fig. 4c). This suggests that DyP activity per cell in liquid cultures containing alizarin is greater than that in cultures without alizarin.

### Time- and pH-dependent decrease in alizarin in liquid culture

**Fig. 5:**
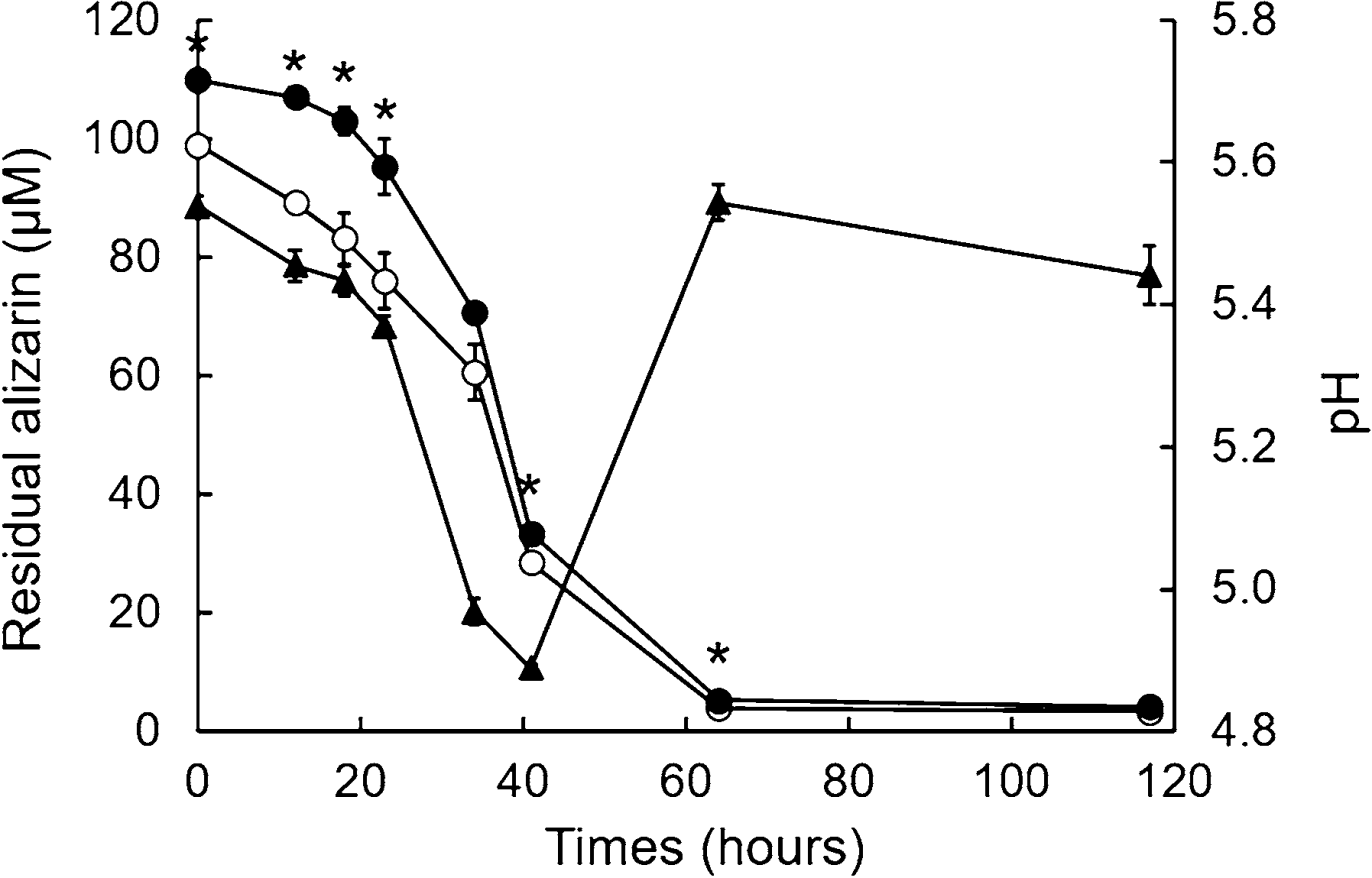
Effect of pH on alizarin degradation by DyP. Time courses of alizarin concentration and pH values in liquid cultures inoculated with 100 μM alizarin. Open and closed circles are residual alizarin concentrations at pH 3.2 (optimum pH for DyP activity) and at unadjusted pH, respectively. Closed triangles denote the time course of pH values. Data are presented as mean ± SD (n = 3; *P < 0.05, unpaired two-tailed t test)

A time course of alizarin concentration in liquid culture indicated little decrease before 24 h and a rapid decrease from 24 to 42 h after inoculation (Fig. 5, closed circles). In addition, the decrease in the pH of the culture broth followed the same time course as the decrease in alizarin concentration (Fig. 5, closed triangles). Given that the optimum pH of DyP from *B.* *adusta* Dec 1 is ~ 3.2, this suggests that alizarin degradation began as the pH in liquid culture crossed a critical threshold value, and increased rapidly as pH fell to within the optimal range (provided DyP is sufficiently expressed). Consistent with this, the alizarin concentration in culture broth adjusted to pH 3.2 decreased at a constant, rapid rate from 0 to 30 h (Fig. 5, opened circles).

## Discussion

DyP degradation activity toward both alizarin and RB19 was similar as shown in Fig. 1, indicating that alizarin is a good substrate for DyP. Therefore, phytoalexins such as alizarin are probable candidate essential substrates. If this were true, *B. adusta* Dec 1 would grow without inhibition in medium containing alizarin. However, our experimental results suggest that the relationship between alizarin degradation and DyP activity during the culture of *B. adusta* is more complicated than this simple picture would suggest.

In plate cultures, increasing the concentration of alizarin caused growth inhibition, but this effect was limited to an early stage of culture (Fig. 2). Interestingly, after this early stage, cells continued to grow without inhibition, suggesting that alizarin is degraded during the early growth of *B.* *adusta*. Similarly, 100 μM alizarin was almost completely degraded before 60 h in liquid shaking culture (Fig. 3). These results are supported by analyses of alizarin in liquid culture (Fig. 5), which showed that alizarin concentration decreased beginning at ~ 24 h. Alizarin showed little degradation prior to 24 h, indicating that DyP was not effective during this period. However, DyP activity per dry cell mass in liquid culture containing 100 μM alizarin was always higher than that of controls during the culture period (Fig. 4c). Notably, DyP activity per dry cell mass in alizarin-treated cultures was greater than that in controls, even at 24 h, suggesting that alizarin had stimulated DyP expression before 24 h (Fig. 4c). These results suggest that alizarin induces greater DyP activity before 24 h in liquid culture, but DyP activity remains below the limit of detection because of the low number of cells prior to 24 h.

Although DyP is apparently induced before 24 h in cultures containing alizarin, alizarin degradation had not yet started. This outcome is attributable to the pH value of the culture, which was unsuitable for DyP activity (Fig. 5). Until 18 h after inoculation, the pH of cultures was maintained above 5.4. Under these conditions, DyP activity toward RB19 is less than 5% of its maximum value (Sugano et al. 2000). On the other hand, from 24 to 40 h in culture, the pH decreased to 4.8–5.3, and DyP exhibited 10–20% of its maximum activity towards RB19 (Sugano et al. 2000). Therefore, if the pH of the culture is below 5.3, this concentration of DyP (0.4 mU/ml) is probably sufficient to degrade alizarin, because DyP showed almost the same degree of degradation activity towards alizarin as it did toward the substrate RB19 (Fig. 1).

The difference in sensitivity of DyP for alizarin between plate cultures and liquid cultures is likely attributable to differences in the diffusion of alizarin. In agarose gels, alizarin diffuses slowly compared with that in solution. Therefore, during degradation by *B.* *adusta*, the concentration of alizarin in the plate becomes less homogeneous than is the case in liquid culture. As a consequence, the degradation rate of alizarin is faster in liquid cultures than in plate cultures for a given starting concentration. In nature, the environment on plates might be more similar to that on trees. Therefore, *B. adusta* would be predicted parasitize trees slowly over time.

Collectively, these results suggest that, upon recognition of an antifungal anthraquinone compound in the environment, *B. adusta* Dec 1 secretes DyP, which then degrades the compounds first before initiating growth. After DyP has sufficiently decomposed the anthraquinone compound to prevent its growth-inhibitory effects, *B. adusta* begins to grow. This relationship accounts for the time lag for the initiation of growth. Thus, we propose that a probable physiological role of the *B. adusta* DyP-type peroxidase is to destroy the antifungal system of plants—a survival mechanism that *B. adusta* exploits in nature to parasitize trees. Notably, this idea is distinct from the concept of DyP involvement in lignin degradation. A *dyp* knockdown or knockout strain would provide a tool for directly testing this idea. Unfortunately, it has proved difficult to construct these strains because *B.* *adusta* has several isozymes of DyP. Moreover, genome-editing techniques for gene knockout in fungi are available only for a subset of yeast and ascomycetes (DiCarlo et al. 2013; Matsu-ura et al. 2015). However, genome editing in basidiomycete was recently reported (Schuster et al. 2016), an encouraging development for the application of these powerful genetic approaches. Although genome editing in *B. adusta* remains challenging because so few applications have been reported, we will attempt to establish a knockout mutant strain in the future. Elucidating the detailed mechanism by which *B.* *adusta* degrades alizarin might pave the way to answering questions regarding interactions between fungi and plants.

## Abbreviations

DyP: dye-decolorizing peroxidase
rDyP: recombinant DyP
LiP: lignin peroxidase
MnP: manganese peroxidase
VP: versatile peroxidase
RB19: Remazol brilliant blue R

## References

[CR1] Ahmad M, Roberts JN, Hardiman EM, Singh R, Eltis LD, Bugg TDH (2011). Identification of DypB from *Rhodococcus jostii* RHA1 as a lignin peroxidase. Biochemistry.

[CR2] Amaral LF, Moriel P, Foglio MA, Mazzola PG (2013). *Caryocar brasiliense* supercritical CO_2_ extract possesses antimicrobial and antioxidant properties useful for personal care products. BMC Complement Altern Med.

[CR3] Berry FH and Lombard FF (1978) Basidiomycetes associated with decay of living oak trees. Forest Service Research Paper NE-413

[CR01] Brown ME, Barros T, Chang MCY (2012). Identification and characterization of a multifunctional dye peroxidase from a lignin-reactive bacterium. ACS Chem Biol.

[CR4] Camarero S, Sarkar S, Ruiz-Dueñas FJ, Martínez MJ, Martínez AT (1999). Description of a versatile peroxidase involved in the natural degradation of lignin that has both manganese peroxidase and lignin peroxidase substrate interaction sites. J Biol Chem.

[CR5] DiCarlo JE, Norville JE, Mali P, Rios X, Aach J, Church GM (2013). Genome engineering in *Saccharomyces cerevisiae* using CRISPR-Cas systems. Nucleic Acids Res.

[CR6] Gomi N, Yoshida S, Matsumoto K, Okudomi M, Konno H, Hisabori T, Sugano Y (2011). Degradation of the synthetic dye amaranth by the fungus *Bjerkandera adusta* Dec 1: inference of the degradation pathway from an analysis of decolorized products. Biodegradation.

[CR7] Jara C, Leyton M, Osorio M, Silva V, Fleming F, Paz M, Madrid A, Mellao M (2017). Antioxidant, phenolic and antifungal profiles of *Acanthus mollis*. Nat Prod Res.

[CR8] Johjima T, Itoh N, Kabuto M, Tokimura F, Nakagawa T, Wariishi H, Tanaka H (1999). Direct interaction of lignin and lignin peroxidase from *Phanerochaete chrysosporium*. Proc Natl Acad Sci USA.

[CR9] Johjima T, Ohkuma M, Kudo T (2003). Isolation and cDNA cloning of novel hydrogen peroxide-dependent phenol oxidase from the basidiomycete *Termitomyces albuminosus*. Appl Microbiol Biotechnol.

[CR10] Kellner H, Luis P, Pecyna MJ, Barbi F, Kapturska D, Kruger D, Zak DR, Marmeisse R, Vandenbol M, Hofrichter M (2014). Widespread occurrence of expressed fungal secretory peroxidases in forest soils. PLoS ONE.

[CR11] Kim SJ, Ishikawa K, Hirai M, Shoda M (1995). Characteristics of a newly isolated fungus, *Geotrichum candidum* Dec 1, which decolorizes various dyes. J Ferment Bioeng.

[CR12] Korripally P, Hunt CG, Houtman CJ, Jones DC, Kitin PJ, Cullen D, Hammel KE (2015). Regulation of gene expression during the onset of ligninolytic oxidation by *Phanerochaete chrysosporium* on spruce wood. Appl Environ Microbiol.

[CR13] Liers C, Bobeth C, Pecyna M, Ullrich R, Hofrichter M (2010). DyP-like peroxidases of the jelly fungus *Auricularia auricula*-*judae* oxidize nonphenolic lignin model compounds and high-redox potential dyes. Appl Microbiol Biotechnol.

[CR14] Liers C, Pecyna MJ, Kellner H, Worrich A, Zorn H, Steffen KT, Hofrichter M, Ullrich R (2013). Substrate oxidation by dye-decolorizing peroxidases (DyPs) from wood- and litter-degrading agaricomycetes compared to other fungal and plant heme-peroxidases. Appl Microbiol Biotechnol.

[CR15] Linde D, Ruiz-Dueñas FJ, Fernández-Fueyo E, Guallar V, Hammel KE, Pogni R, Martínez AT (2015). Basidiomycete DyPs: genomic diversity, structural–functional aspects, reaction mechanism and environmental significance. Arch Biochem Biophys.

[CR16] Manojlovic NT, Solujic S, Sukdolak S, Milosev M (2005). Antifungal activity of *Rubia tinctorum*, *Rhamnus frangula* and *Caloplaca cerina*. Fitoterapia.

[CR17] Matsu-ura T, Baek M, Kwon J, Hong C (2015). Efficient gene editing in *Neurospora crassa* with CRISPR technology. Fungal Biol Biotechnol.

[CR18] Ogola HJO, Kamiike T, Hashimoto N, Ashida H, Ishikawa T, Shibata H, Sawa Y (2009). Molecular characterization of a novel peroxidase from the cyanobacterium *Anabaena* sp. strain PCC 7120. Appl Environ Microbiol.

[CR19] Pollegioni L, Tonin F, Rosini E (2015). Lignin-degrading enzymes. FEBS J.

[CR20] Rahmanpour R, Bugg TDH (2013). Assembly in vitro of *Rhodococcus jostii* RHA1 encapsulin and peroxidase DypB to form a nanocompartment. FEBS J.

[CR21] Roberts JN, Singh R, Grigg JC, Murphy MEP, Bugg TDH, Eltis LD (2011). Characterization of dye-decolorizing peroxidases from *Rhodococcus jostii* RHA1. Biochemistry.

[CR23] Salvachúa D, Prieto A, Martínez AT, Martínez MJ (2013). Characterization of a novel dye-decolorizimg peroxidase (DyP)-type enzyme from *Irpex lacteus* and its application in enzymatic hydrolysis of wheat straw. Appl Environ Microbiol.

[CR24] Santos A, Mendes S, Brissos V, Martins LO (2014). New dye-decolorizing peroxidases from *Bacillus subtilis* and *Pseudomonas putida* MET94: towards biotechnological applications. Appl Microbiol Biotechnol.

[CR25] Scheibner M, Hulsdau B, Zelena K, Nimtz M, de Boer L, Berger RG, Zorn H (2008). Novel peroxidases of *Marasmius scorodonius* degrade beta-carotene. Appl Microbiol Biotechnol.

[CR26] Schuster M, Schweizer G, Reissmann S, Kahmann R (2016). Genome editing in *Ustilago maydis* using the CRISPR-Cas system. Fungal Genet Biol.

[CR27] Shintani N, Sugano Y, Shoda M (2002). Decolorization of kraft pulp lignin bleaching effluent by a newly isolated fungus, *Geotrichum candidum* Dec 1. J Wood Sci.

[CR28] Singh R, Grigg JC, Armstrong Z, Murphy MEP, Eltis LD (2012). Distal heme pocket residues of B-type dye-decolorizing peroxidase: arginine but not aspartate is essential for peroxidase activity. J Biol Chem.

[CR29] Sugano Y (2009). DyP-type peroxidases comprise a novel heme peroxidase family. Cell Mol Life Sci.

[CR30] Sugano Y, Nakano R, Sasaki K, Shoda M (2000). Efficient heterologous expression in *Aspergillus oryzae* of a unique dye-decolorizing peroxidase, DyP, of *Geotrichum candidum* Dec 1. Appl Environ Microbiol.

[CR31] Sugano Y, Matsushima Y, Shoda M (2006). Complete decolorization of the anthraquinone dye Reactive blue 5 by the concerted action of two peroxidases from *Thanatephorus cucumeris* Dec 1. Appl Microbiol Biotechnol.

[CR32] Sugano Y, Muramatsu R, Ichiyanagi A, Sato T, Shoda M (2007). DyP, a unique dye-decolorizing peroxidase, represents a novel heme peroxidase family: ASP171 replaces the distal histidine of classical peroxidases. J Biol Chem.

[CR33] Sugano Y, Matsushima Y, Tsuchiya K, Aoki H, Hirai M, Shoda M (2009). Degradation pathway of an anthraquinone dye catalyzed by a unique peroxidase DyP from *Thanatephorus cucumeris* Dec 1. Biodegradation.

[CR34] Sugawara K, Nishihashi Y, Narioka T, Yoshida T, Morita M, Sugano Y (2017). Characterization of a novel DyP-type peroxidase from *Streptomyces avermitilis*. J Biosci Bioeng.

[CR35] Wariishi H, Dunford HB, MacDonald ID, Gold MH (1989). Manganese peroxidase from the lignin-degrading basidiomycete *Phanerochaete chrysosporium*. Transient state kinetics and reaction mechanism. J Biol Chem.

[CR36] Wijnsma R, Go JT, van Weerden IN, Harkes PA, Verpoorte R, Baerheim SA (1985). Anthraquinones as phytoalexins in cell and tissue cultures of *Cinchona* spec. Plant Cell Rep.

[CR37] Yoshida T, Sugano Y (2015). A structural and functional perspective of DyP-type peroxidase family. Arch Bioch Biophys.

